# Supplementary material for: Ensemble-based enzyme design can recapitulate the effects of laboratory directed evolution in silico
Source: Nat Commun. 2020 Sep 23;11:4808. doi: 10.1038/s41467-020-18619-x (PMC7511930; doi:10.1038/s41467-020-18619-x)
Supplement: Supplementary file 1 — Supplementary Information [file 41467_2020_18619_MOESM1_ESM.pdf]

Supplementary Information

Ensemble-based enzyme design can recapitulate the effects of laboratory directed evolution *in silico*

Aron Broom[1,4], Rojo V. Rakotoharisoa[1,4], Michael C. Thompson[2,3], Niayesh Zarifi[1], Erin Nguyen[1], Nurzhan Mukhametzhanov[1], Lin Liu[2], James S. Fraser[2] & Roberto A. Chica[1,*]

[1] Department of Chemistry and Biomolecular Sciences, University of Ottawa, 10 Marie Curie, Ottawa, Ontario, K1N 6N5, Canada.

[2] Department of Bioengineering and Therapeutic Science, University of California, San Francisco, San Francisco, California 94158, United States.

[3] Department of Chemistry and Chemical Biology, University of California, Merced, California, United States

[4] These authors contributed equally: Aron Broom, Rojo V. Rakotoharisoa.

[*] correspondence: rchica@uottawa.ca

Supplementary Table 1. Amino-acid sequences of HG-series Kemp eliminases

**Enzyme**	**# mutations from HG3**	**Sequence**[a]
**HG3**	–	MAEAAQSVDQLIKARGKVYFGVATDQNRLTTGKNAAIIQADFGMVWPENS<br>MKWDATEPSQGNFNFAGADYLVNWAQQNGKLIGGGMLVWHSQLPSWVSSI<br>TDKNTLTNVMKNHITTLMTRYKGKIRAWDVVGEAFNEDGSLRQTVFLNVI<br>GEDYIPIAFQTARAADPNAKLYIMDYNLDSASYPKTQAIVNRVKQWRAAG<br>VPIDGIGSQTHLSAGQGAGVLQALPLLASAGTPEVSILMLDVAGASPTDY<br>VNVVNACLNVQSCVGITVFGVADPDSWRASTTPLLFDGNFNPKPAYNAIV<br>QDLQQGSIEGRGHHHHHH
**HG3.3b**	6	MAEAAQS**I**DQLIKARGKVYFGVATDQNRLTTGKNAAIIQADFGMVWPENS<br>M**H**WDATEPSQGNFNFAGADYLVNWAQQNGKLIGGG**C**LVWH**RD**LPSWVSSI<br>TDKNTLTNVMKNHITTLMTRYKGKIR**N**WDVVGEAFNEDGSLRQTVFLNVI<br>GEDYIPIAFQTARAADPNAKLYIMDYNLDSASYPKTQAIVNRVKQWRAAG<br>VPIDGIGSQTHLSAGQGAGVLQALPLLASAGTPEVSILMLDVAGASPTDY<br>VNVVNACLNVQSCVGITVFGVADPDSWRASTTPLLFDGNFNPKPAYNAIV<br>QDLQQGSIEGRGHHHHHH
**HG3.7**	7	MAEAAQS**I**DQLIKARGKVYFGVATDQNRLTTGKNAAII**K**ADFGMVWPENS<br>M**Q**WDATEPSQGNFNFAGADYLVNWAQQNGKLIGGG**C**LVWH**RH**LPSWVSSI<br>TDKNTLTNVMKNHITTLMTRYKGKIR**N**WDVVGEAFNEDGSLRQTVFLNVI<br>GEDYIPIAFQTARAADPNAKLYIMDYNLDSASYPKTQAIVNRVKQWRAAG<br>VPIDGIGSQTHLSAGQGAGVLQALPLLASAGTPEVSILMLDVAGASPTDY<br>VNVVNACLNVQSCVGITVFGVADPDSWRASTTPLLFDGNFNPKPAYNAIV<br>QDLQQGSIEGRGHHHHHH
**HG3.14**	12	MAEAAQS**I**DQLIKARGKVYFGVATDQNRLTTGKNAAII**K**ADFGMVWPENS<br>M**Q**WDATEPSQGNFNFAGADYLVNWAQQNGKLIG**A**G**C**LVWHS**H**LPSWVSSI<br>TDKNTL**I**NVMKNHITTLMTRYKGKIR**T**WDVVGEAFNEDGSLRQ**N**VFLNVI<br>GEDYIPIAFQTARAADPNAKLYIMDYNLDSASYPKTQAIVNRVKQWRAAG<br>VPIDGIGSQ**M**HLSAGQGAGVLQALPLLASAGTPEVSILMLDVAGASPTDY<br>VNVVNACLNVQSCVGITVFGVADPDSWRAS**S**TPLLFDGNFNPKPAYNAIV<br>Q**N**LQQGSIEGRGHHHHHH
**HG3.17**	17	MAEAAQS**I**DQLIKARGKVYFGVATDQNRLTTGKNAAII**K**ADFGMVWPE**E**S<br>M**Q**WDATEPSQGNFNFAGADYLVNWAQQNGKLIG**A**G**C**LVWH**NF**LPSWVSSI<br>TDKNTL**I**NVMKNHITTLMTRYKGKIR**T**WDVVGEAFNEDGSLRQ**N**VFLNVI<br>GEDYIPIAFQTARAADPNAKLYIMDYNLDSASYPKTQAIVNRVKQWRAAG<br>VPIDGIGSQ**M**HLSAGQGAGVLQALPLLASAGTPEVSILMLDVAGASPTDY<br>VNVVNACLNVQSCVGITV**M**GVADPDS**AF**AS**S**TPLLFDGNFNPKPAYNAIV<br>Q**N**LQQGSIEGRGHHHHHH
**HG4**	8	MAEAAQSVDQLIKARGKVYFGVATDQNRLTTGKNAAIIQADFGMVWPENS<br>M**Q**WDATEPSQGNFNFAGADYLVNWAQQNGKLIG**A**G**C**LVWHS**F**LPSWVSSI<br>TDKNTLTNVMKNHITTLMTRYKGKIR**T**WDVVGEAFNEDGSLRQTVFLNVI<br>GEDYIPIAFQTARAADPNAKLYIMDYNLDSASYPKTQAIVNRVKQWRAAG<br>VPIDGIGSQTHLSAGQGAGVLQALPLLASAGTPEVSILMLDVAGASPTDY<br>VNVVNACLNVQSCVGITV**M**GVADPDS**AF**ASTTPLLFDGNFNPKPAYNAIV<br>QDLQQGSIEGRGHHHHHH

[a] Mutations from HG3 are highlighted in bold. All sequences contain a His-tag at the C-terminus.

Supplementary Table 2. Crystallization conditions

Enzyme [a]	6NT [b]	pH	Protein (mg mL^{-1})	$(NH_4)_2SO_4$ (M)
**HG3**	(–)	4.6	4	1.8
	(+)	4.6	4	1.8
**HG3.3b**	(–)	5.4	5	2.0
	(+)	5.4	5	2.0
**HG3.7**	(–)	4.0	6	1.6
	(+)	5.0	12	2.0
**HG3.14**	(–)	4.5	3	1.6
	(+)	5.0	3	2.4
**HG3.17**	(–)	5.0	5	1.6
	(+)	5.0	10	1.6
**HG4**	(–)	4.6	10	1.6
	(+)	4.6	10	1.6

[a] All enzymes were crystallized in 100 mM sodium acetate buffer at the indicated pH.
[b] 6-nitrobenzotriazole (6NT) was dissolved in pure dimethyl sulfoxide (DMSO) and added to crystallization solution at a final concentration of 5 mM (5% DMSO).

Supplementary Table 3. Crystallographic data and refinement statistics for room-temperature structures (277 K)

	HG3		HG3.3b		HG3.7		HG3.14		HG3.17		HG4	
**6NT**	(–)	(+)	(–)	(+)	(–)	(+)	(–)	(+)	(–)	(+)	(–)	(+)
PDB ID	5RG4	5RGA	5RG5	5RGB	5RG6	5RGC	5RG7	5RGD	5RG8	5RGE	5RG9	5RGF
**Data collection** [a]												
Resolution (Å)	41.52–1.99	55.15–1.89	37.09–1.62	48.16–1.44	60.34–1.35	79.81–1.39	79.83–1.47	48.94–1.40	46.17–1.73	38.46–1.77	79.99–1.47	41.50–1.40
Space group	$P2_12_12_1$	$P2_12_12_1$	$P2_12_12_1$	$P2_12_12_1$	$P2_12_12_1$	$P2_12_12_1$	$P2_12_12_1$	$P2_12_12_1$	$P2_12_12_1$	$P2_12_12_1$	$P2_12_12_1$	$P2_12_12_1$
Cell params.												
a b c (Å)	76.14 79.97 99.06	76.24 79.88 99.05	76.23 80.03 98.91	76.24 79.92 98.71	76.20 79.85 98.81	76.26 79.81 98.72	76.31 79.83 99.11	76.32 79.81 98.94	51.35 58.17 92.35	50.90 57.98 95.69	76.29 79.99 98.98	75.97 77.99 98.03
α β γ (°)	90 90 90	90 90 90	90 90 90	90 90 90	90 90 90	90 90 90	90 90 90	90 90 90	90 90 90	90 90 90	90 90 90	90 90 90
Molecules per asymm. unit	2	2	2	2	2	2	2	2	1	1	2	2
R_{pim}	0.125 (0.375)	0.067 (0.332)	0.047 (0.407)	0.043 (0.540)	0.029 (0.246)	0.029 (0.359)	0.029 (0.372)	0.046 (0.391)	0.088 (0.734)	0.101 (0.606)	0.048 (0.550)	0.028 (0.375)
$CC_{1/2}$	0.974 (0.679)	0.994 (0.745)	0.997 (0.690)	0.998 (0.524)	0.998 (0.826)	0.999 (0.681)	0.999 (0.586)	0.998 (0.697)	0.996 (0.587)	0.990 (0.499)	0.997 (0.609)	0.999 (0.743)
I/σI	3.7 (1.0)	6.8 (1.0)	8.9 (1.0)	8.9 (1.1)	13.0 (2.3)	12.6 (1.3)	13.6 (1.4)	9.4 (1.4)	8.8 (1.1)	5.5 (1.0)	8.2 (1.1)	29.4 (3.5)
Completeness (%)	100.0 (100.0)	100.0 (100.0)	100.0 (100.0)	99.8 (99.8)	99.0 (91.8)	100.0 (99.6)	99.8 (99.2)	100.0 (100.0)	100.0 (99.5)	99.5 (99.6)	99.7 (99.6)	99.8 (99.0)
Multiplicity	6.4 (6.5)	12.7 (9.3)	12.9 (10.9)	6.4 (6.4)	6.3 (4.7)	12.7 (10.0)	18.5 (10.4)	13.0 (13.5)	16.5 (6.3)	6.5 (6.1)	5.8 (5.9)	17.3 (13.0)
Wilson B-factor (Å^2)	17.21	17.58	14.72	14.85	11.43	13.07	13.31	14.34	14.08	13.30	14.07	15.40
# reflections (total unique)	42219	51523	118990	113916	131151	121461	103204	80367	29625	27504	103164	114707
**Refinement**												
R work/free	0.1767/ 0.2164	0.1482/ 0.1863	0.1421/ 0.1665	0.1360/ 0.1665	0.1271/ 0.1412	0.1255/ 0.1409	0.1319/ 0.1480	0.1329/ 0.1518	0.1397/ 0.1615	0.1501/ 0.1794	0.1434/ 0.1633	0.1317/ 0.1497
No. atoms												
Protein	4754	5017	5261	5376	5661	5703	5407	5066	2398	2387	5164	5180
Ligand	–	24	–	24	–	24	–	24	–	12	–	24
Water	424	483	463	498	543	527	511	496	267	228	474	488
Averaged B-factors (Å^2)												
Protein	25.85	25.65	24.74	22.83	18.78	21.11	23.73	24.92	20.75	20.94	25.38	25.40
Ligand	–	29.10	–	20.17	–	15.46	–	18.52	–	15.41	–	17.54
Water	33.56	35.97	35.69	36.07	33.62	35.81	37.51	37.24	33.28	31.83	36.97	37.27
RMSD												
bond lengths (Å)	0.005	0.008	0.004	0.005	0.008	0.008	0.008	0.005	0.004	0.003	0.004	0.004
bond angles (°)	0.831	0.910	0.784	0.880	1.029	0.986	0.973	0.835	0.731	0.631	0.773	0.822
MolProbity clashscore	2.97	3.09	3.35	4.67	3.89	2.02	2.32	2.26	1.26	1.26	2.91	2.70

[a] Highest resolution shell is shown in parentheses.

Supplementary Table 4. Amino-acid positions optimized during computational design of HG4

Ligand placement [a]	Repacking [b]
16, 17, 21, 42, 44, 46, 47, 79, 81, 83, 84, 87, 90, 125, 130, 170, 172, 207, 209, 234, 236, 237, 239, 265, 267, 275, 276	V16, Y17, A21, M42, W44, E46, N47, Q50, L79, G81, A82, G83, C84, W87, F90, T125, D127, G130, Y170, M172, Q207, H209, S234, L236, M237, D239, T265, M267, A275, F276

[a] Positions that were mutated to Gly during ligand placement. Catalytic residues D127 and Q50 were allowed to sample alternate rotamers. All other residues were kept fixed.
[b] Positions and amino-acid types that were allowed to sample alternate rotamers during repacking. All other residues were kept fixed.

Supplementary Table 5. Geometric definitions for generation of transition-state poses off the side chains of catalytic residues

Contact	Type	Atom 1 [a]	Atom 2 [a]	Atom 3 [a]	Atom 4 [a]	Values [b]
**Asp127**	Distance	OD1 or OD2	**H3**			1.0, 1.2, 1.5
	Angle	CG	OD1 or OD2	**H3**		112, 117, 122
	Angle	OD1 or OD2	**H3**	**C3**		159, 164, 169, 174, 179
	Torsion	CB	CG	OD1 or OD2	**H3**	0, 5, 10, 170, 175, 180
	Torsion	CG	OD1 or OD2	H3	**C3**	170, 175, 180, 185, 190
	Torsion	OD1 or OD2	**H3**	**C3**	**N2**	0, 5, 170, 175, 180
**Gln50**	Distance	1HE2 or 2HE2	**O1**			1.2, 1.5, 1.7, 1.9, 2.1, 2.3
	Angle	NE2	1HE2 or 2HE2	**O1**		145, 148, 151, 154, 157
	Angle	2HE2	**O1**	**N2**		120, 125, 135, 145, 155
	Torsion	CD	NE2	1HE2 or 2HE2	**O1**	115, 120, 135, 140, 145
	Torsion	NE2	1HE2 or 2HE2	**O1**	**N2**	180, 190, 200, 210, 220, 230
	Torsion	1HE2 or 2HE2	**O1**	**N2**	**C3**	150, 160, 170, 180, 190, 200

[a] Atoms in bold are from the transition state. All other atoms are from the catalytic residues.
[b] Distance measurements given in Å, all others in degrees.

Supplementary Table 6. Geometric constraints used to define catalytic contacts during HG4 computational design

Contact	Type	Atom 1 [a]	Atom 2 [a]	Atom 3 [a]	Atom 4 [a]	Min [b]	Max [b]
**Asp127**	Distance	OD1 or OD2	**H3**			1.0 (1.0)	1.6 (1.6)
	Angle	CG	OD1 or OD2	**H3**		109 (109)	131 (131)
	Angle	OD1 or OD2	**H3**	**C3**		159 (159)	180 (180)
	Torsion	CB	CG	OD1 or OD2	**H3**	–21 (–21)	21 (21)
**Gln50**	Distance	1HE2 or 2HE2	**O1**			1.2 (1.2)	2.3 (3.2)
	Angle	NE2	1HE2 or 2HE2	**O1**		147 (130)	157 (180)
	Angle	1HE2 or 2HE2	**O1**	**N2**		120 (111)	140 (151)
	Torsion	1HE2 or 2HE2	**O1**	**N2**	**C3**	160 (130)	200 (200)

[a] Atoms in bold are from the transition state. All other atoms are from the catalytic residues.
[b] Distance measurements given in Å, all others in degrees. Values in parentheses are for the repacking step, while the others are for ligand placement.

Supplementary Table 7. Codon-optimized genes of HG-series Kemp eliminases

Enzyme	DNA sequence
**HG3**	ATGGCGGAGGCGGCGCAGAGCGTGGACCAACTGATCAAGGCGCGTGGCAAGGTTTACTTTGGCGTGGCGACCGACCAGAATCGTCTGACCACC<br>GGCAAGAACGCGGCGATCATTCAGGCGGACTTCGGCATGGTGTGGCCGGAGAACAGCATGAAATGGGATGCGACCGAACCGAGCCAGGGTAAC<br>TTCAACTTTGCGGGCGCGGACTACCTGGTTAACTGGGCGCAGCAAAACGGCAAGCTGATCGGTGGCGGTATGCTGGTGTGGCACAGCCAGCTG<br>CCGAGCTGGGTTAGCAGCATTACCGATAAGAACACCCTGACCAACGTGATGAAAAACCACATCACCACCCTGATGACCCGTTATAAGGGTAAA<br>ATTCGTGCGTGGGACGTGGTTGGCGAGGCGTTCAACGAAGATGGCAGCCTGCGTCAGACCGTGTTTCTGAACGTTATCGGCGAGGACTACATC<br>CCGATTGCGTTTCAGACCGCGCGTGCGGCGGACCCGAACGCGAAACTGTACATCATGGACTATAACCTGGATAGCGCGAGCTATCCGAAGACC<br>CAGGCGATTGTGAACCGTGTTAAACAATGGCGTGCGGCGGGTGTGCCGATTGATGGTATTGGTAGCCAGACCCATCTGAGCGCGGGTCAGGGT<br>GCGGGCGTTCTGCAAGCGCTGCCGCTGCTGGCGAGCGCGGGTACCCCGGAAGTGAGCATTCTGATGCTGGATGTTGCGGGTGCGAGCCCGACC<br>GATTACGTTAACGTGGTTAACGCGTGCCTGAACGTGCAAAGCTGCGTTGGTATTACCGTGTTTGGTGTTGCGGACCCGGATAGCTGGCGTGCG<br>AGCACCACCCCGCTGCTGTTCGATGGCAACTTTAACCCGAAGCCGGCGTATAACGCGATTGTTCAAGATCTGCAACAGGGTAGCATCGAGGGT<br>CGTGGTCATCATCATCATCATCACTAA
**HG3.3b**	ATGGCGGAGGCGGCGCAGAGCATCGACCAACTGATTAAGGCGCGTGGTAAAGTGTACTTCGGCGTTGCGACCGATCAGAACCGTCTGACCACC<br>GGCAAGAACGCGGCGATCATTCAAGCGGACTTTGGCATGGTGTGGCCGGAGAACAGCATGCACTGGGATGCGACCGAACCGAGCCAGGGTAAC<br>TTCAACTTTGCGGGCGCGGACTATCTGGTTAACTGGGCGCAGCAAAACGGTAAACTGATCGGTGGCGGTTGCCTGGTGTGGCACCGTGACCTG<br>CCGAGCTGGGTTAGCAGCATTACCGATAAGAACACCCTGACCAACGTGATGAAAAACCACATCACCACCCTGATGACCCGTTACAAGGGTAAA<br>ATTCGTAACTGGGACGTGGTTGGCGAGGCGTTCAACGAAGATGGCAGCCTGCGTCAGACCGTGTTTCTGAACGTTATCGGCGAAGACTACATC<br>CCGATTGCGTTTCAAACCGCGCGTGCGGCGGACCCGAACGCGAAGCTGTACATCATGGACTATAACCTGGATAGCGCGAGCTATCCGAAGACC<br>CAGGCGATTGTGAACCGTGTTAAACAATGGCGTGCGGCGGGTGTGCCGATTGATGGTATTGGTAGCCAGACCCATCTGAGCGCGGGTCAGGGT<br>GCGGGCGTTCTGCAGGCGCTGCCGCTGCTGGCGAGCGCGGGTACCCCGGAAGTGAGCATTCTGATGCTGGATGTTGCGGGTGCGAGCCCGACC<br>GATTACGTGAACGTGGTTAACGCGTGCCTGAACGTGCAGAGCTGCGTTGGTATTACCGTGTTTGGTGTTGCGGACCCGGATAGCTGGCGTGCG<br>AGCACCACCCCGCTGCTGTTCGACGGTAACTTTAACCCGAAACCGGCGTATAACGCGATCGTTCAAGATCTGCAGCAAGGCAGCATTGAAGGT<br>CGTGGCCACCACCACCACCACCACTAA
**HG3.7**	ATGGCAGAAGCGGCGCAGAGCATCGACCAACTGATTAAGGCGCGTGGCAAGGTTTACTTTGGCGTGGCGACCGACCAGAACCGTCTGACCACC<br>GGCAAGAACGCGGCGATCATTAAAGCGGACTTCGGCATGGTGTGGCCGGAGAACAGCATGCAGTGGGATGCGACCGAACCGAGCCAGGGTAAC<br>TTCAACTTTGCGGGCGCGGACTACCTGGTTAACTGGGCGCAGCAAAACGGCAAGCTGATCGGTGGCGGTTGCCTGGTGTGGCACCGTCACCTG<br>CCGAGCTGGGTTAGCAGCATTACCGATAAGAACACCCTGACCAACGTGATGAAAAACCACATCACCACCCTGATGACCCGTTATAAGGGTAAA<br>ATTCGTAACTGGGACGTGGTTGGCGAGGCGTTCAACGAAGATGGCAGCCTGCGTCAGACCGTGTTTCTGAACGTTATCGGCGAGGACTACATC<br>CCGATTGCGTTTCAGACCGCGCGTGCGGCGGACCCGAACGCGAAACTGTACATCATGGACTATAACCTGGATAGCGCGAGCTATCCGAAGACC<br>CAGGCGATTGTGAACCGTGTTAAACAATGGCGTGCGGCGGGTGTGCCGATTGATGGTATTGGTAGCCAGACCCATCTGAGCGCGGGTCAGGGT<br>GCGGGCGTTCTGCAAGCGCTGCCGCTGCTGGCGAGCGCGGGTACCCCGGAAGTGAGCATTCTGATGCTGGATGTTGCGGGTGCGAGCCCGACC<br>GATTACGTTAACGTGGTTAACGCGTGCCTGAACGTGCAAAGCTGCGTTGGTATTACCGTGTTTGGTGTTGCGGACCCGGATAGCTGGCGTGCG<br>AGCACCACCCCGCTGCTGTTCGATGGCAACTTTAACCCGAAGCCGGCGTATAACGCGATTGTTCAAGATCTCCAACAGGGTAGCATCGAGGGT<br>CGTGGTCACCATCATCACCACCATTAA
**HG3.14**	ATGGCGGAAGCGGCGCAGAGCATCGACCAACTGATCAAGGCGCGTGGCAAGGTGTACTTTGGCGTGGCGACCGACCAGAACCGTCTGACCACC<br>GGCAAGAACGCGGCGATCATTAAAGCGGACTTCGGTATGGTGTGGCCGGAGAACAGCATGCAGTGGGATGCGACCGAACCGAGCCAAGGCAAC<br>TTCAACTTTGCGGGTGCGGACTACCTGGTTAACTGGGCGCAGCAAAACGGCAAGCTGATTGGTGCGGGTTGCCTGGTGTGGCACAGCCACCTG<br>CCGAGCTGGGTTAGCAGCATCACCGATAAGAACACCCTGATTAACGTGATGAAAAACCACATCACCACCCTGATGACCCGTTATAAGGGCAAA<br>ATTCGTACCTGGGACGTGGTTGGCGAGGCGTTCAACGAAGATGGTAGCCTGCGTCAGAACGTGTTTCTGAACGTTATCGGCGAGGACTACATC<br>CCGATTGCGTTTCAGACCGCGCGTGCGGCGGACCCGAACGCGAAACTGTACATTATGGACTATAACCTGGATAGCGCGAGCTATCCGAAGACC<br>CAGGCGATCGTGAACCGTGTTAAACAGTGGCGTGCGGCGGGCGTGCCGATTGATGGTATTGGCAGCCAAATGCACCTGAGCGCGGGTCAAGGT<br>GCGGGTGTTCTGCAAGCGCTGCCGCTGCTGGCGAGCGCGGGTACCCCGGAAGTGAGCATTCTGATGCTGGATGTTGCGGGTGCGAGCCCGACC<br>GATTACGTTAACGTGGTTAACGCGTGCCTGAACGTGCAAAGCTGCGTTGGCATCACCGTGTTTGGTGTTGCGGACCCGGATAGCTGGCGTGCG<br>AGCAGCACCCCGCTGCTGTTCGATGGTAACTTTAACCCGAAGCCGGCGTATAACGCGATCGTTCAAAATCTGCAACAAGGTAGCATCGAGGGT<br>CGTGGTCATCATCATCATCATCACTAA
**HG3.17**	ATGGCGGAGGCGGCGCAGAGCATCGACCAACTGATTAAGGCGCGTGGCAAAGTGTACTTCGGTGTTGCGACCGATCAGAACCGTCTGACCACC<br>GGCAAGAACGCGGCGATCATTAAAGCGGACTTTGGTATGGTGTGGCCGGAGGAAAGCATGCAGTGGGATGCGACCGAACCGAGCCAAGGCAAC<br>TTCAACTTTGCGGGTGCGGACTATCTGGTTAACTGGGCGCAGCAAAACGGCAAGCTGATCGGTGCGGGCTGCCTGGTGTGGCACAACTTCCTG<br>CCGAGCTGGGTTAGCAGCATCACCGATAAGAACACCCTGATTAACGTGATGAAAAACCACATCACCACCCTGATGACCCGTTACAAGGGCAAA<br>ATTCGTACCTGGGACGTGGTTGGCGAGGCGTTCAACGAAGATGGTAGCCTGCGTCAGAACGTGTTTCTGAACGTTATCGGCGAGGACTACATC<br>CCGATTGCGTTCCAGACCGCGCGTGCGGCGGACCCGAACGCGAAACTGTACATTATGGACTATAACCTGGATAGCGCGAGCTATCCGAAGACC<br>CAGGCGATCGTGAACCGTGTTAAACAGTGGCGTGCGGCGGGTGTGCCGATTGACGGTATTGGCAGCCAGATGCACCTGAGCGCGGGTCAAGGT<br>GCGGGTGTTCTGCAAGCGCTGCCGCTGCTGGCGAGCGCGGGTACCCCGGAAGTGAGCATTCTGATGCTGGATGTTGCGGGTGCGAGCCCGACC<br>GATTACGTGAACGTGGTTAACGCGTGCCTGAACGTGCAGAGCTGCGTTGGCATCACCGTGATGGGTGTTGCGGACCCGGATAGCGCGTTTGCG<br>AGCAGCACCCCGCTGCTGTTCGATGGCAACTTTAACCCGAAGCCGGCGTATAACGCGATTGTTCAAAACCTGCAGCAAGGTAGCATCGAAGGC<br>CGTGGTCACCACCACCACCACCACTAA
**HG4**	ATGGCGGAGGCGGCGCAGAGCGTGGACCAACTGATCAAGGCGCGTGGCAAGGTTTACTTTGGCGTGGCGACCGACCAGAATCGTCTGACCACC<br>GGCAAGAACGCGGCGATCATTCAGGCGGACTTCGGCATGGTGTGGCCGGAGAACAGCATGCAGTGGGATGCGACCGAACCGAGCCAGGGTAAC<br>TTCAACTTTGCGGGCGCGGACTACCTGGTTAACTGGGCGCAGCAAAACGGCAAGCTGATCGGTGCGGGTTGCCTGGTGTGGCACAGCTTTCTG<br>CCGAGCTGGGTTAGCAGCATTACCGATAAGAACACCCTGACCAACGTGATGAAAAACCACATCACCACCCTGATGACCCGTTATAAGGGTAAA<br>ATTCGTACGTGGGACGTGGTTGGCGAGGCGTTCAACGAAGATGGCAGCCTGCGTCAGACCGTGTTTCTGAACGTTATCGGCGAGGACTACATC<br>CCGATTGCGTTTCAGACCGCGCGTGCGGCGGACCCGAACGCGAAACTGTACATCATGGACTATAACCTGGATAGCGCGAGCTATCCGAAGACC<br>CAGGCGATTGTGAACCGTGTTAAACAATGGCGTGCGGCGGGTGTGCCGATTGATGGTATTGGTAGCCAGACCCATCTGAGCGCGGGTCAGGGT<br>GCGGGCGTTCTGCAAGCGCTGCCGCTGCTGGCGAGCGCGGGTACCCCGGAAGTGAGCATTCTGATGCTGGATGTTGCGGGTGCGAGTCCGACC<br>GATTACGTTAACGTGGTTAACGCGTGCCTGAACGTGCAAAGCTGCGTTGGTATTACCGTGATGGGTGTTGCGGACCCGGATAGCGCGTTTGCG<br>AGCACCACCCCGCTGCTGTTCGATGGCAACTTTAACCCGAAGCCGGCGTATAACGCGATTGTTCAAGATCTGCAACAGGGTAGCATCGAGGGT<br>CGTGGTCATCATCATCATCATCACTAA

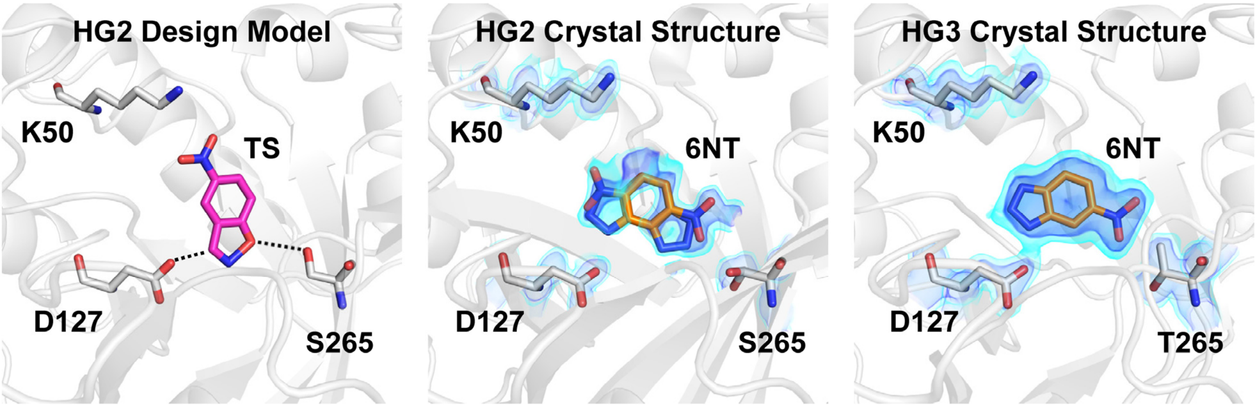

Supplementary Figure 1. HG2 is the direct precursor to HG3. For the crystal structures of HG2 and HG3, the 2Fo-Fc map is shown in volume representation at two contour levels: 0.5 eÅ^{-3} and 1.5 eÅ^{-3} in light and dark blue, respectively. HG2 was designed *in silico* to stabilize the transition state (TS) via catalytic contacts (dashed lines) with the D127 base and the S265 hydrogen bond donor (Left panel). However, its crystal structure (PDB ID: 3NYD) showed that the transition state analogue 6-nitrobenzotriazole (6NT) was bound in two alternate orientations (Middle panel). In the catalytically productive pose, the acidic N–H bond of 6NT that mimics the cleavable C–H bond of the substrate is located within H-bonding distance to the carboxylate oxygen of D127, and the nitro group is close to S265 (found to adopt two conformations in the crystal structure). In the catalytically non-productive pose, 6NT is flipped, which positions its nitro group closer to K50, and its acidic N–H bond far from the side chain of D127. To increase activity, Privett *et al.* introduced the S265T mutation into HG2 post-design to reduce the active-site conformational heterogeneity that was observed by molecular dynamics analysis, leading to the higher-activity variant HG3 (Right panel). Of note, no density for the non-productive binding pose of 6NT was observed in the HG3 crystal structure reported here.

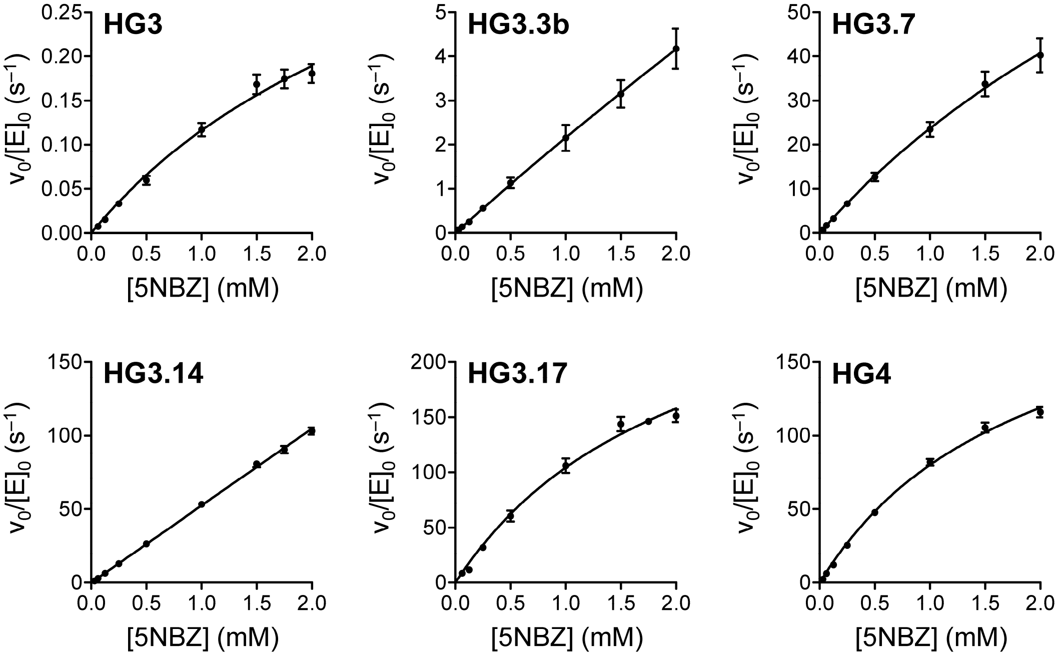

Supplementary Figure 2. Steady-state kinetics. Michaelis–Menten plots of normalized initial rates as a function of 5-nitrobenzisoxazole (5NBZ) concentrations are shown. Data represent the average of six or nine individual replicate measurements from two or three independent protein batches, with error bars indicating the SEM (n = 2 independent experiments for HG3, HG3.3b, HG3.7 and HG3.17, n = 3 independent experiments for HG3.14 and HG4, mean ± SEM in all cases). Saturation was not achieved for any enzyme at the substrate's solubility limit (2 mM). Therefore, only k_{cat}/K_M values are reported on Table 1, and these values were calculated using linear regression of rates measured at the three or four lowest substrate concentrations. Under these conditions, [S] << K_M, and the Michaelis-Menten equation becomes $v_0 = (k_{cat}/K_M)[E_0][S]$. Source data are provided as a source data file.

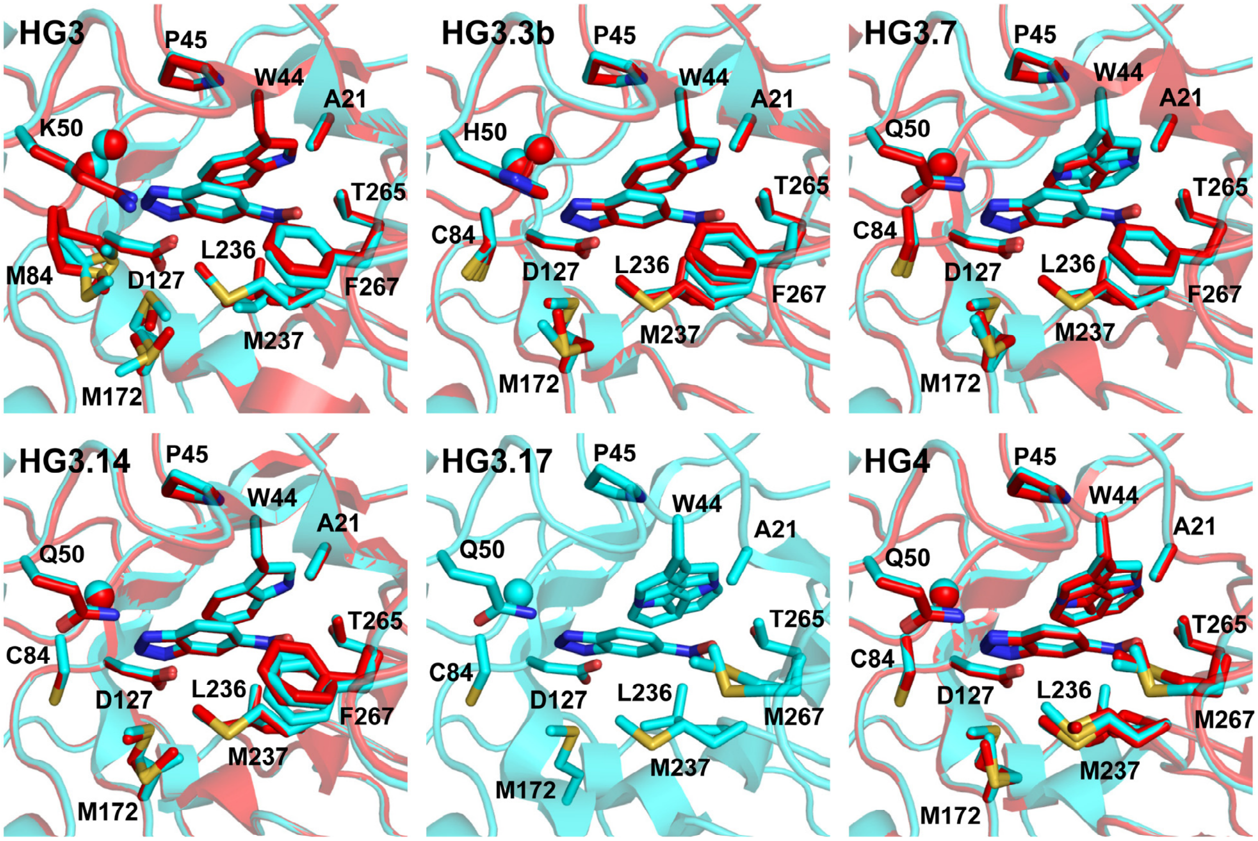

Supplementary Figure 3. 6NT-binding pocket. Overlay of chains A (cyan) and B (red) showing residues forming the 6NT-binding pocket in all HG-series Kemp eliminases. For HG3.17, only chain A is shown as its asymmetric unit contained a single polypeptide chain. In all cases, 6NT is bound in the middle of the barrel. Spheres indicate alpha carbons of Gly83 (two conformers, *cis*/*trans*, are observed for peptide bond between Gly83 and Met/Cys84 in HG3 and HG3.3b).

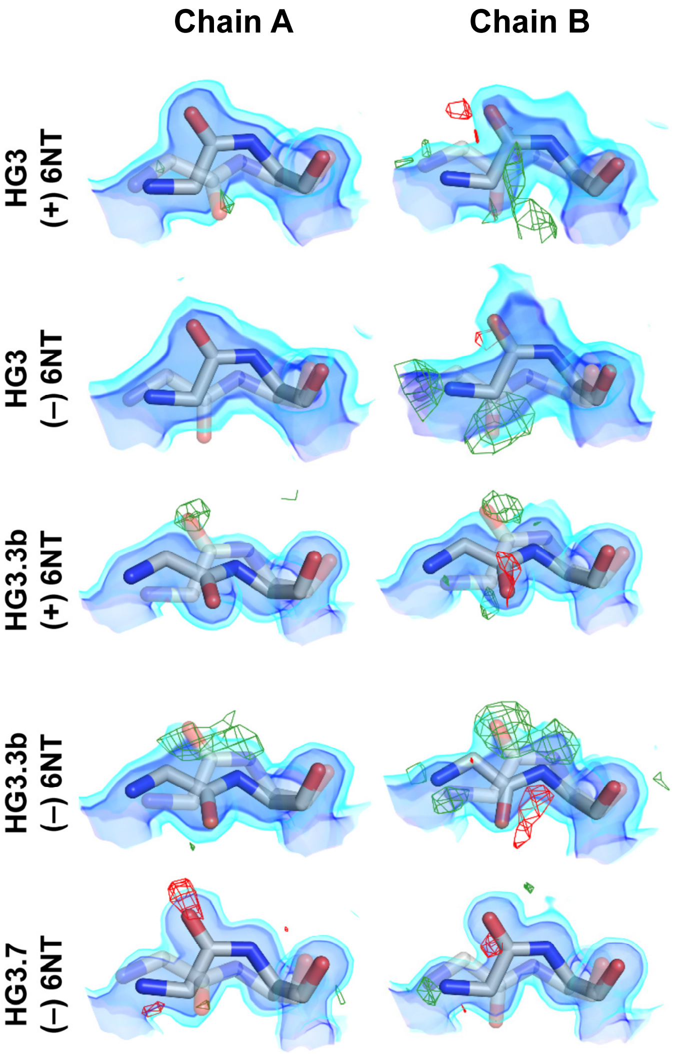

Supplementary Figure 4. Difference density increases around the 83–84 peptide bond after refinement in the absence of minor conformer. The peptide bond between residues 83 and 84 adopts both *cis* and *trans* conformations in HG3, HG3.3b, and HG3.7. To confirm the presence of the alternate peptide, the structure was re-refined in the absence of the minor conformer (transparent sticks), resulting in difference density for one or both chains in the asymmetric unit of HG3 and HG3.3b. A similar effect was also seen for HG3.7 in the unbound state, although the difference features corresponding to the minor conformation were weaker in comparison to HG3 and HG3.3b.

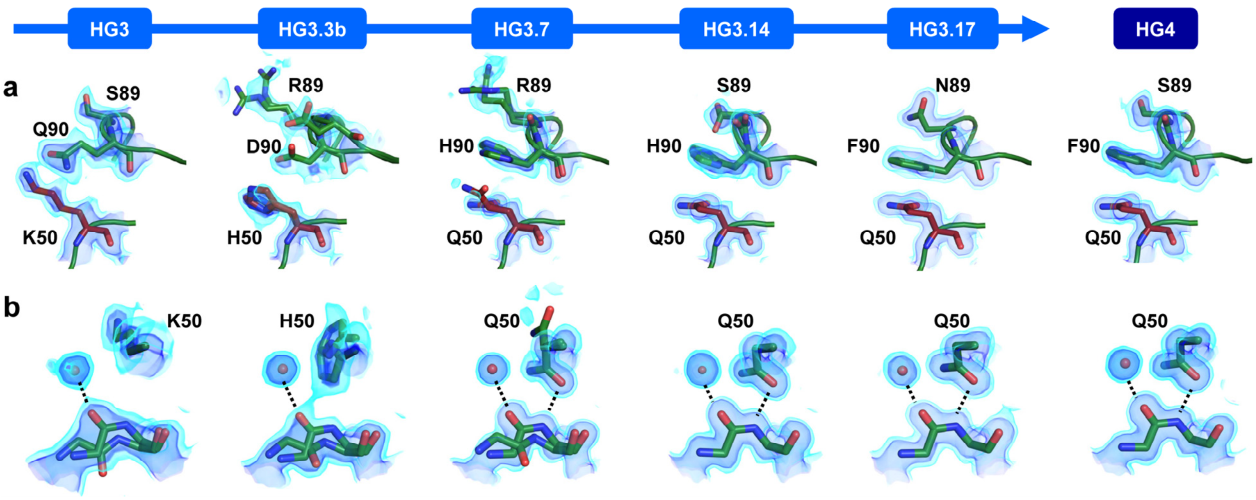

Supplementary Figure 5. Crystal structures of HG-series Kemp eliminases in the unbound state. In all cases, only atoms from chain A are shown. The 2Fo-Fc maps are shown in volume representation at two contour levels: 0.5 eÅ^{-3} and 1.5 eÅ^{-3} in light and dark blue, respectively. (a) Conformational changes to loop formed by residues 87–90 over the course of the evolutionary trajectory. (b) The peptide bond between residues 83 and 84 adopts both *cis* and *trans* conformations in HG3, HG3.3b, and HG3.7, but only the *cis* conformation in the higher activity variants. Ordered water molecules are shown as red spheres, and hydrogen bonds as dashed lines.

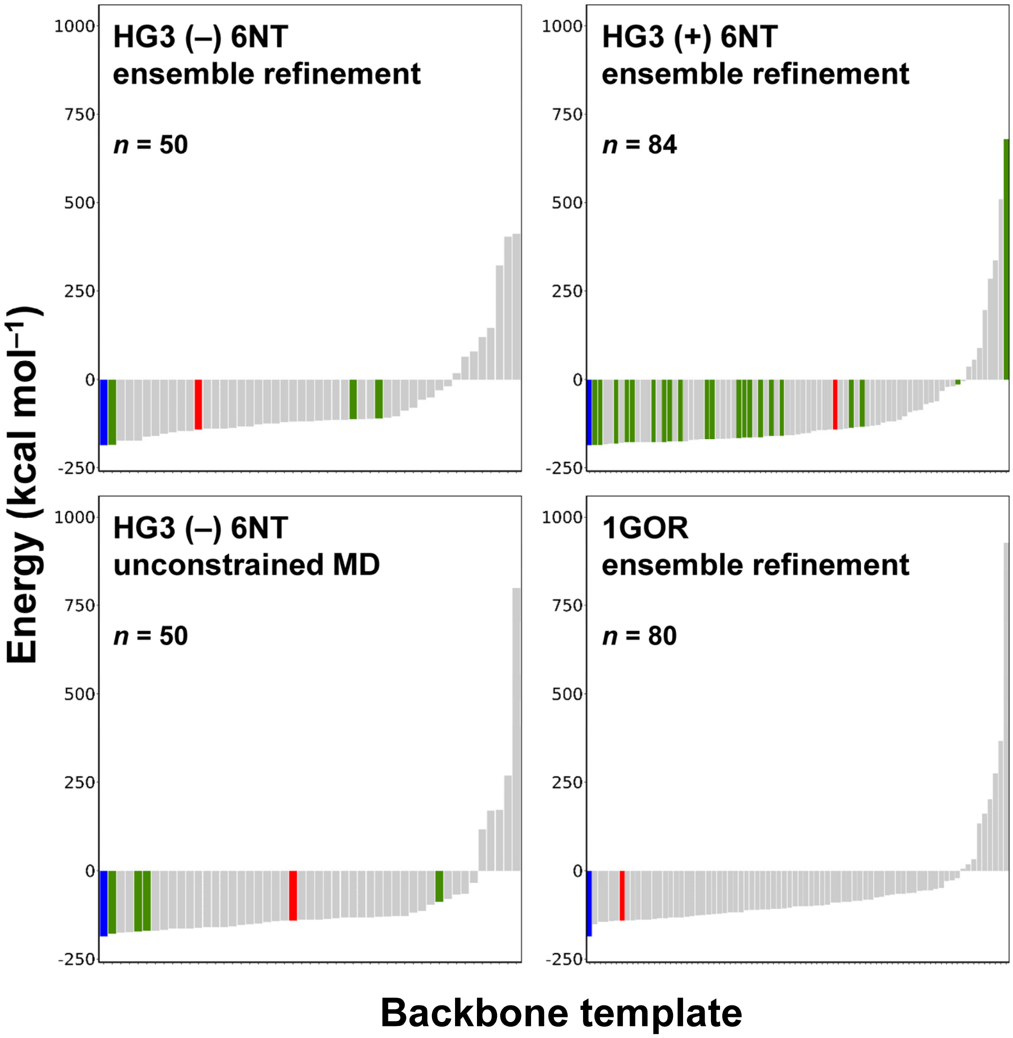

Supplementary Figure 6. Energy of HG4 design models generated on various backbone templates. Rotamers for the HG4 sequence and its associated transition state binding pose were optimized (Methods) on individual backbone templates (bars). These ensembles of backbone templates were generated using molecular dynamics (MD) constrained or not by the diffraction data. In all cases, *n* indicates the total number of templates in the ensemble. Green bars indicate templates that yielded design models with transition-state binding poses within 0.7 Å root-mean-square deviation from the crystallographic binding pose. Blue and red bars indicate design models obtained from the HG4 with bound 6NT (–186.4 kcal/mol) or 1GOR (–141.6 kcal/mol) crystal structures, respectively. All HG3-derived ensembles yielded at least one HG4 design model with an accurate transition-state binding pose (i.e., root-mean-square deviation < 0.7 Å) and a more favorable energy than that obtained on the 1GOR template. However, the ensemble prepared from the HG3 (+) 6NT data outperforms all others: it yielded the lowest energy model and the highest number of models with accurate transition-state binding poses (21 out of 84).

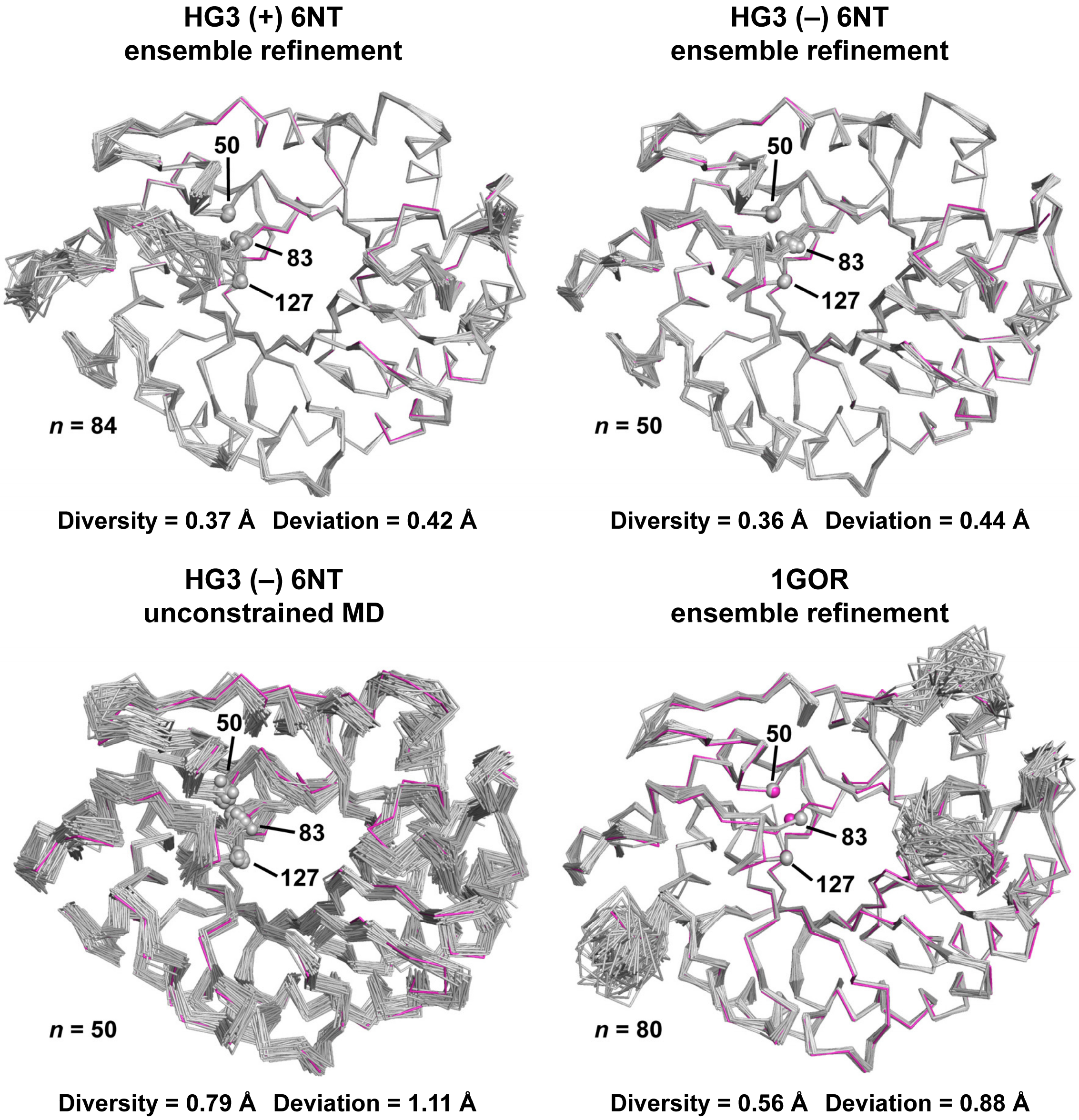

Supplementary Figure 7. Backbone ensembles. The crystal structure of HG4 (+) 6NT (magenta) is superimposed with members of ensembles (grey) generated by ensemble refinement or unconstrained molecular dynamics (MD) starting from the HG3 (+) 6NT, HG3 (–) 6NT, or 1GOR crystal structures. Spheres indicate alpha carbons of key active-site residues. Ensemble properties, such as the average root-mean-square backbone coordinate deviation between pairs of ensemble members (diversity) or average root-mean-square backbone coordinate deviation from the HG4 (+) 6NT crystal structure (deviation) are indicated. In all cases, *n* indicates the number of templates per ensemble. Several templates from HG3-derived ensembles enable recapitulation of the crystallographic transition-state binding pose (Figure 4e,f,h and Supplementary Figure 6) due to the ability of the residue 83 backbone to sample conformations that eliminate steric clashes with this ligand, and that are similar to the one observed in the HG4 (+) 6NT crystal structure (i.e., there is overlap of magenta and grey spheres at position 83). This is not the case for the 1GOR-derived ensemble, in which the position of the residue 83 backbone does not shift substantially, preventing it from adopting a conformation necessary to allow the crystallographic transition-state binding pose found in the HG4 (+) 6NT crystal structure (i.e., there is no overlap of magenta and grey spheres at position 83). Of note, the ensemble generated by unconstrained MD (bottom left) samples a range of conformations with higher diversity and deviation than the corresponding one generated from ensemble refinement (top right), and with evenly-distributed conformational heterogeneity throughout the structure. By contrast, conformational heterogeneity in ensembles generated using crystallographic restraints is unevenly distributed, being higher in specific structural elements.
