# Supplementary material for: Ensemble-based enzyme design can recapitulate the effects of laboratory directed evolution in silico
Source: Nat Commun. 2020 Sep 23;11:4808. doi: 10.1038/s41467-020-18619-x (PMC7511930; doi:10.1038/s41467-020-18619-x)
Supplement: Supplementary file 3 — Description of Additional Supplementary Files [file 41467_2020_18619_MOESM3_ESM.pdf]

Description of Additional Supplementary Files

File Name: Supplementary Software
Description: This file contains the Triad scripts used within this study, as well as the attribute file specifying force field parameters for the transition state.
